# African League Against Rheumatism (AFLAR) preliminary recommendations on the management of rheumatic diseases during the COVID-19 pandemic

**DOI:** 10.1007/s10067-020-05355-2

**Published:** 2020-09-02

**Authors:** Richard Oluyinka Akintayo, Rachid Bahiri, Yasser El Miedany, Hakeem Olaosebikan, Asgar Ali Kalla, Adewale Olukayode Adebajo, Angela Nyangore Migowa, Samy Slimani, Ouma Devi Koussougbo, Ben Abdelghani Kawther, Akpabio Akanimo Akpabio, Imad Ghozlani, Dzifa Dey, Waleed A. Hassan, Nimmisha Govind, Kavita Makan, Abdelgaffar Mohamed, Eugene Kalman Genga, Mohamed Khattry Ahmed Ghassem, Mohamed Mortada, Wafa Hamdi, Moudjib O. Wabi, Mohammed Tikly, Madeleine Ngandeu-Singwe, Christian Scott

**Affiliations:** 1 Rheumatology Department, Dumfries and Galloway Royal Infirmary, Cargenbridge, Dumfries, UK; 2 Department of Rheumatology, El Ayachi Hospital, Medical University, Rabat, Morocco; 3 Rheumatology and Rehabilitation Department, Ain Shams University, Cairo, Egypt; 4 Lagos State University College of Medicine and Lagos State University Teaching Hospital, Ikeja, Lagos, Nigeria; 5 Department of Medicine, University of Cape Town, Cape Town, South Africa; 6 Faculty of Medicine, Dentistry and Health, University of Sheffield, Sheffield, UK; 7 Aga Khan University Hospital Nairobi Kenya, Nairobi, Kenya; 8 Atlas Clinic of Rheumatology, 05000 Batna, Algeria; 9 Victoria Hospital, Republic of Mauritius, Quatre Bornes, Mauritius; 10 Rheumatology Department, Mongi Slim Hospital, Tunis faculty of Medicine, Tunis EL Manar University, Tunis, Tunisia; 11 University of Uyo Teaching Hospital, Uyo, Akwa Ibom Nigeria; 12 Department of Rheumatology, Military Hospital, University Ibn Zohr, Agadir, Morocco; 13 University of Ghana Medical School, Korlebu Teaching Hospital, Accra, Ghana; 14 Rheumatology and Rehabilitation, Benha University, Banha, Egypt; 15 Division of Rheumatology, Chris Hani Baragwanath Academic Hospital, University of the Witwatersrand, Johannesburg, South Africa; 16 Chris Hani Baragwanath Academic Hospital University of the Witwatersrand, Johannesburg, South Africa; 17 Rheumatology, King Abdulla Hospital, Bisha, Kingdom of Saudi Arabia, Bisha, Saudi Arabia; 18 University of Nairobi, Nairobi, Kenya; 19 Rheumatology Department, Military Hospital Mohammed V, Rabat, Morocco; 20 Rheumatology and Rehabilitation Faculty of Medicine, Zagazig University, Zagazig, Egypt; 21 Department of rheumatology, Kassab Institute UR17SP04 Faculty of Medicine of Tunis, Tunis El Manar University, Tunis, Tunisia; 22 Rhumatologie, Doctorant Épidémiologie Clinique et Sciences Médico – Chirurgicales, Polyclinique Apkapka, Cotonou, Bénin; 23 Chris Hani Baragwanath Academic Hospital, University of the Witwatersrand, Johannesburg, South Africa; 24 Faculty of Medicine and Biomedical Sciences, University of Yaounde, Yaounde, Cameroon; 25 University of Cape Town, Cape Town, South Africa; 26 School of Health and Life Sciences, Glasgow Caledonian University, Cowcaddens Road, Glasgow,, G4 0BA UK

**Keywords:** African rheumatology, COVID-19, DMARD, Rheumatic and musculoskeletal diseases, Rheumatology

## Abstract

**Objectives:**

To develop recommendations for the management of rheumatic and musculoskeletal diseases (RMDs) during the COVID-19 pandemic.

**Method:**

A task force comprising of 25 rheumatologists from the 5 regions of the continent was formed and operated through a hub-and-spoke model with a central working committee (CWC) and 4 subgroups. The subgroups championed separate scopes of the clinical questions and formulated preliminary statements of recommendations which were processed centrally in the CWC. The CWC and each subgroup met by several virtual meetings, and two rounds of voting were conducted on the drafted statements of recommendations. Votes were online-delivered and recommendations were pruned down according to predefined criteria. Each statement was rated between 1 and 9 with 1–3, 4–6 and 7–9 representing disagreement, uncertainty and agreement, respectively. The levels of agreement on the statements were stratified as low, moderate or high according to the spread of votes. A statement was retired if it had a mean vote below 7 or a ‘low’ level of agreement.

**Results:**

A total of 126 initial statements of recommendations were drafted, and these were reduced to 22 after the two rounds of voting.

**Conclusions:**

The preliminary statements of recommendations will serve to guide the clinical practice of rheumatology across Africa amidst the changing practices and uncertainties in the current era of COVID-19. It is recognized that further updates to the recommendations will be needed as more evidence emerges.

**Table:** 

**Key Points** *• AFLAR has developed preliminary recommendations for the management of RMDs in the face of the COVID-19 pandemic.* *• COVID-19 is an unprecedented experience which has brought new concerns regarding the use of some disease-modifying anti-rheumatic drugs (DMARDs), and these recommendations seek to provide guidelines to the African rheumatologists.* *• Hydroxychloroquine shortage has become rampart across Africa as the drug is being used as prophylaxis against COVID-19 and this may necessitate a review of treatment plan for some patients with RMDs.* *• Breastfeeding should continue for as long as possible if a woman is positive for SARS-CoV-2 as there is currently no evidence that the infection can be transmitted through breast milk.*

## Introduction

Since the advent of the COVID-19 pandemic and the rapid spread of the disease across the globe, the practice of rheumatology has found itself in the middle of crucial decision making and widespread service disruption. Across Africa, the impact of the pandemic is even more noticeable as there is a wide disparity in the availability and quality of healthcare services in the different countries and regions of the continent [1]. Recommendations and practice standards have changed rapidly in the face of the pandemic, and the potential roles of a number of disease-modifying anti-rheumatic drugs (DMARDs) in the management of COVID-19 have come to the fore.

The leadership of the African League Against Rheumatism (AFLAR) commissioned a task force to develop recommendations for the management of rheumatic and musculoskeletal diseases (RMDs) in the unprecedented situation of a continent faced with COVID-19. The task force had a very short time to deliver the project. However, the relatively delayed spread of the severe acute respiratory syndrome coronavirus 2 (SARS-CoV-2) across the continent a while after other continents such as Asia, North America and Europe allowed for some body of evidence to have been developed and recommendations made by other international rheumatology bodies which became available for the appraisal of the African team.

## Methods

### Mandate

The scope of the proposed recommendations was to address clinical questions encountered on a daily basis by the practicing rheumatologists in Africa in the complex situation where COVID-19 redefines clinical needs and standards. Recommendations were proposed to cover the management of RMDs among adult and paediatric patients in the context of COVID-19. The task excludes recommendations for the management of individual RMDs or the treatment of COVID-19. The lead and membership of the central working committee (CWC) and subsequently the full membership list of the larger task force were commissioned by the president of AFLAR to produce the recommendations within predefined deadlines.

### Structure of the task force

A hub-and-spoke model was adopted with the task force spreading out from the CWC formed by the leads of the four subgroups as well as the AFLAR president, an executive of the Pediatric African League Against Rheumatism (PAFLAR) and 2 members of the COVID-19 African Rheumatology Research Group. The leads of the subgroups were assigned and delegated with the tasks and the broad themes of each subgroup were as follows: subgroup 1, risk assessment and prevention of SARS-CoV-2 infection among patients with RMDs; subgroup 2, use of usual rheumatological treatments in patients at risk of SARS-CoV-2 infection; subgroup 3, treatment of patients with RMDs after exposure to known case of COVID-19; and subgroup 4, management of RMDs in patients infected with SARS-CoV-2. The subgroup leads were given the mandate to nominate the membership of their subgroups which were to be drawn from all five regions of the continent. The complete task force was made up of 25 practicing rheumatologists of which the scope of practice was adult only in 10, adult and paediatrics in 11 and paediatrics only in 4. In conjunction with other paediatric rheumatologists on the task force, the PAFLAR executive on the CWC led an independent review and representation of the paediatric-specific recommendations to the CWC.

### Timeline

The CWC was set up on June 8, and the first virtual meeting was held on June 10 by video conferencing. The subsequent processes and timelines were agreed upon. The return of full preliminary list of task force membership was attained on June 12, and the submission of the first set of statements was completed by June 17. The first round of voting was held on June 20, while the second round commenced on June 21. Harmonization of statements and approval of final statements were completed on June 29.

### Background guideline review

Published guidelines and recommendations of international rheumatology bodies outside Africa on management of RMDs in the context of COVID-19 were reviewed. This includes the recently published guidelines of the European League Against Rheumatism (EULAR), American College of Rheumatology (ACR), the Australian Rheumatology Association (ARA), the Asia Pacific League of Associations for Rheumatology (APLAR) and the National institute for Health and Care Excellence (NICE) of the UK [2–6]. Relevant aspects were drawn for consideration as one arm of the recommendations classed as ‘general’. A second arm was designated ‘Africa-Specific’, and it includes clinical questions and recommendations that are specific to the settings and prevailing situations in Africa. Each subgroup was tasked with developing their clinical questions and recommendations along these two broad divisions.

### Target audience

The primary target audience are practicing rheumatologists in Africa. This includes rheumatologists managing adult, paediatric or both categories of patients. The focus excludes patients, other physicians or policy-makers. However, recommendations covering advice to patients which are to be delivered by the rheumatologist are included.

### Literature review

It was agreed that there is currently an insufficient body of evidence to conduct a meaningful systematic literature review. In addition, the short working timeline available to the task force precluded further delays. However, a limited non-systematic literature review was undertaken to support the largely expert opinion-based process. This was conducted between the 4 subgroup leads and harmonized in the CWC.

### Processing of clinical questions and recommendations

Each subgroup was tasked to develop the clinical questions according to the theme of their subgroup and make preliminary recommendations to be submitted to the central working committee towards the first round of votes. The four subgroups turned in a total of one hundred and twenty-six preliminary statements of recommendation which were processed centrally. Overlaps, duplication and out-of-scope recommendations were removed or amended, and the first list for the first round of voting was agreed upon by the CWC. At the end of the first round of voting, there were twenty-four statements of recommendation. These were processed further into the second round of voting which led to the final agreement on twenty-two statements.

### Voting process

Live online-delivered voting was done in two stages that were strictly time limited. All members of the task force were invited to participate and were pre-informed of the time of opening and closure of each round of votes. Unique access links were sent out, and anonymous votes were gathered and processed. Comments on re-phrasing, potential ambiguity, unidentified overlaps and so on were gathered regarding each statement at the same time in the voting process. Only the members of the task force who treat either both adults and children or children only voted on the statements that are specific to paediatric rheumatology.

### Rating

Each statement was rated between 1 and 9 with 1 being ‘complete disagreement’ and 9 being ‘complete agreement’. Generally, 1–3, 4–6 and 7–9 represented disagreement, uncertainty and agreement, respectively. There was no requirement to vote on all statements, and the members were encouraged to abstain if they felt that a statement fell outside their area of expertise. Therefore, an ‘uncertainty’ vote represented ‘unconvinced about the veracity of the recommendation’ and excludes ‘unsure of the current best practice in this context’. All statements were allowed for the entry of comments which were reviewed by the CWC after each round of voting. In the second round of votes, the members were further urged to leave comments wherever they voted a disagreement. This enabled the panel to identify an instance of misinterpretation of statement and invalidate the vote on that statement.

### Predefined parameters

The levels of agreement on each statement of recommendation were defined as ‘high’ if after the second round of votes, all votes on a statement fell into the agreement bracket; ‘low’ if at least one vote fell into each of the disagreement and uncertainty bracket or two or more fell into disagreement bracket; and ‘moderate’ for all other combinations. A statement was retired if it had a mean vote below 7 or a ‘low’ level of agreement.

## Results

A total of twenty-two recommendations were generated, and a breakdown is presented in Table 1. Below is a brief explanation of each statement and the considerations leading to their formation.

**Table 1:** Breakdown of statements of recommendations

S/N	Statements	Mean rank ±SD	Level of agreement
1	Statement 1: There is currently no evidence to say that patients with RMDs or on DMARDs are more prone to contracting SARS-CoV-2; therefore, the normal medications for various rheumatic diseases should be continued as indicated	8.8 ± 0.4	H
2	Statement 2: Patients should be routinely advised to observe regular infection-prevention practices against SARS-CoV-2 such as social distancing, frequent hand washing and wearing of face covering in public places	9.0 ± 0	H
3	Statement 3: Patients should be advised to avoid using medicinal products with unproven efficacy against COVID-19. Such products could include herbal preparations, orthodox medicines, various supplements and complementary medications	8.7 ± 0.8	M
4	Statement 4: In a patient without COVID-19, it is safe to initiate or maintain the use of NSAIDs for RMDs where indicated	8.6 ± 0.5	H
5	Statement 5: In a patient without COVID-19, oral or parenteral glucocorticoids may be used as indicated at the appropriate dose including pulse doses where necessary	8.9 ± 0.4	H
6	Statement 6: Anti-malarials such as HCQ and CQ do not protect against SARSCoV-2, and as such, patients on HCQ or CQ for RMDs should be advised to observe necessary precautions to prevent infection	8.8 ± 0.5	H
7	Statement 7: Where HCQ scarcity is being experienced, rheumatologists may consider using alternative csDMARDs for patients with rheumatoid arthritis	8.4 ± 0.9	M
8	Statement 8: Where possible, the use of subcutaneous formulations of bDMARDs and bsDMARDs should be considered instead of IV infusions to limit patients’ attendance to the hospital	8.8 ± 0.5	H
9	Statement 9: ACEIs and ARBs should not be routinely discontinued in patients with RMDs at risk of COVID-19	8.2 ± 1.2	M
10	Statement 10: Patients with stable RMDs should have their vaccination against influenza and pneumococci updated	8.8 ± 0.5	H
11	Statement 11: Rheumatologists should reduce patients’ hospital attendances by considering less frequent blood monitoring for DMARDs among stable patients, longer prescription periods and virtual clinics	8.9 ± 0.4	H
12	Statement 12: RMD patients on glucocorticoids who have been exposed to SARS-CoV-2 should continue their glucocorticoids at the lowest effective dose for maintaining disease control	8.6 ± 0.66	H
13	Statement 13: In patients who have been exposed to SARS-CoV-2 but are asymptomatic, continue anti-osteoporosis treatments including vitamin D and calcium supplementation	8.7 ± 0.5	H
14	Statement 14: Following exposure to SARS-CoV-2 infection, continue PJP prophylaxis in a patient who is already on it	8.3 ± 1.0	M
15	Statement 15: Regardless of recent exposure to SARS-CoV-2, ACEIs and ARBs may be initiated or increased in a patient with RMD if indicated	8.1 ± 1.2	M
16	Statement 16: Regardless of recent exposure to SARS-CoV-2, intra-articular steroid administration may be undertaken if indicated with the use of appropriate personal protective equipment (PPE)	8.8 ± 0.4	H
17	Statement 17: In an RMD patient with confirmed or presumed COVID-19, ongoing glucocorticoid treatment should not be stopped but should be maintained at the lowest effective dose	8.5 ± 0.5	H
18	Statement 18: In an RMD patient with confirmed or presumed COVID-19, unless specifically indicated, routine stoppage of NSAIDs, analgesics, ACEIs or ARB is not required.	8.4 ± 0.6	H
19	Statement 19: Each rheumatology service should consider providing a helpline number to patients to use or for their doctors to use to seek rheumatological advice should they develop COVID-19.	8.8 ± 0.5	H
20	Statement 20: There is currently no proof that SARS-CoV-2 infection is transmitted through breastfeeding, thus breastfeeding may be continued while observing personal hygiene precautions if a lactating woman has tested positive for SARS-CoV-2 infection.	7.4 ± 1.8	M
21	Statement 21: In the event that a lactating mother with COVID-19 is too unwell to breastfeed the child, expressed breast milk or formula feeds should be considered and these should be prepared using hygienic practices as recommended by government and regulatory bodies of the given region.	7.0 ± 1.7	M
22	Statement 22: Among paediatric patients with suspected SARS-CoV-2 infection who have negative nasopharyngeal PCR test, PCR test on stool samples should be considered to help confirm the diagnosis.	7.0 ± 1.7	M

*SD* standard deviation, *DMARD* disease-modifying anti-rheumatic drug, *RMD* rheumatic and musculoskeletal disease, *SARS-CoV-2* severe acute respiratory syndrome coronavirus 2, *NSAID* non-steroidal anti-inflammatory drug, *COVID-19* coronavirus disease 2019, *HCQ* hydroxychloroquine, *CQ* chloroquine, *csDMARD* conventional synthetic disease-modifying anti-rheumatic drug, *bDMARD* biologic disease-modifying anti-rheumatic drug, *bsDMARD* biosimilar disease-modifying anti-rheumatic drug, *ACEI* angiotensin-converting enzyme inhibitor, *ARB* angiotensin receptor blocker, *PCR* polymerase chain reaction, *H* high, *M* moderate

### Statement 1

*There is currently no evidence to say that patients with RMDs or on DMARDs are more prone to contracting SARS-CoV-2; therefore, the normal medications for various rheumatic diseases should be continued as indicated.*

The risk factors for getting infected with the novel coronavirus are similar for patients with RMDs as for the general populace, and these include older age and co-morbidities such as hypertension and diabetes [2, 7]. Patients with RMDs have not been identified to be more prone to developing COVID-19 than people without RMDs, and this seems to be similar for the previous outbreaks of earlier corona viruses such as the severe acute respiratory syndrome coronavirus (SARS-CoV) and the Middle East respiratory syndrome coronavirus (MERS-CoV) [8, 9]. Mixed results have been reported with patients on prednisolone of more than 10 mg per day as well as disease-modifying agents such as anti-TNF and other biologics. Also, conventional DMARDs and NSAIDs have not been identified to increase the odds of infection or hospitalization [10]. So far, it would appear that there is no definite evidence to date that ongoing treatments for RMDs confer added risks of infection with SARS-CoV-2 [11, 12].

### Statement 2

*Patients should be routinely advised to observe regular infection-prevention practices against SARS-CoV-2 such as social distancing, frequent hand washing and wearing of face covering in public places*.

Standard infection-prevention practices should be frequently communicated and reinforced at every opportunity to the patients according to the recommendations in each member country of AFLAR [2, 3]. This was widely agreed upon by all members of the task force in recognition that there are some differences between the national policies of the member countries against COVID-19. Components of the rheumatologist’s advice should include regular and adequate hand washing, social distancing, avoiding touching the face and coughing into the elbow [13]. Other specific infection-prevention advice may be relevant to the individual patient scenario or the prevailing local guidelines.

### Statement 3

*Patients should be advised to avoid using medicinal products with unproven efficacy against COVID-19. Such products could include herbal preparations, orthodox medicines, various supplements and complementary medications.*

It was recognized that a long list can be made of various unproven remedies which have attained different levels of fame in the media since the onset of COVID-19 pandemic. While this is not limited to Africa, the continent certainly has its fair share of promoted orthodox, herbal and complementary medicines of unproven efficacy against COVID-19. Some of these have been shown to be associated with some risks in earlier reports [14, 15].

### Statement 4

*In a patient without COVID-19, it is safe to initiate or maintain the use of NSAIDs for RMDs where indicated.*

For many patients who depend on regular NSAID use for managing their RMDs, routine withdrawal of such treatment may be counterproductive to the goal of care. This is particularly noteworthy as exposure to NSAIDs has not been shown to increase the risk of infection with SARS-CoV-2. Whereas NSAIDs should generally be used in the lowest effective dose, the use in patients who go on to develop COVID-19 should be based on a favourable risk-benefit ratio [3, 16]. Certainly, there is currently no evidence-backed contraindication to the use of NSAIDs in the patient who is at risk of COVID19 [17].

### Statement 5

*In a patient without COVID-19, oral or parenteral glucocorticoids may be used as indicated at the appropriate dose including pulse doses where necessary.*

It is recognized that the fear of glucocorticoid use in the era of COVID-19 may expose patients with organ-threatening diseases such as the vasculitides and aggressive connective tissue diseases to greater risks if appropriate interventions using the required immunosuppressive agents are not promptly adopted. Undoubtedly, patients with these conditions may face short-term risks of major organ damage or mortality without appropriate treatment [18]. Therefore, in the context of these severe diseases, the appropriate dose of glucocorticoids including high and pulse doses may be unavoidable [3].

### Statement 6

*Anti-malarials such as HCQ and CQ do not protect against SARSCoV-2, and as such, patients on HCQ or CQ for RMDs should be advised to observe necessary precautions to prevent infection.*

Despite the in vitro anti-viral activities of HCQ and CQ which have been reported in various studies, these anti-malaria drugs have not been shown to prevent SAS-CoV-2 infection and patients with SLE on long-term treatment with anti-malarial drugs have not been shown to be spared from the infection or developing severe diseases [19]. The task force recognized that there have been unsubstantiated suggestions at the beginning of the pandemic that the widespread use of anti-malarial drugs in Africa may be contributing to the delayed spread of the virus on the continent.

### Statement 7

*Where HCQ scarcity is being experienced, rheumatologists may consider using alternative csDMARDs for patients with rheumatoid arthritis.*

The recent pan-African survey through the network of the COVID-19 African Rheumatology Study Group has shown that shortage of HCQ was being experienced in more than 60% of rheumatologists’ services across the continent [1]. Therefore, for patients with rheumatoid arthritis, it was agreed that alternative DMARDs may be considered where appropriate. The task force recognized that some patients on triple therapy with stable disease may be able to continue with two csDMARDs without suffering flare ups.

### Statement 8

*Where possible, the use of subcutaneous formulations of bDMARDs and bsDMARDs should be considered instead of IV infusions to limit patients’ attendance to the hospital.*

The availability of biologic and biosimilar agents is highly varied across the continent of Africa. Many countries do not have access to any biosimilar drugs, and in others, the subcutaneous formulations of existing specific intravenously administered biologics are not available. It was agreed that rheumatologists will have to consider the options within the limit of the available agents in their country. However, the driving goal of limiting in-patient hospital visits for infusion administration should be pursued wherever possible.

### Statement 9

*ACEIs and ARBs should not be routinely discontinued in patients with RMDs at risk of COVID-19.*

Whereas there is no evidence to suggest that ACEIs or ARBs increase the risk of SARS-CoV-2 infection, a large population-based study has revealed that the prevalence of use of these agents among patients with COVID19 is higher than in the general populace [20]. This is probably indicative of a higher prevalence of cardiovascular co-morbidities such as hypertension among patients with clinical and severe COVID-19, and this category of patients is also more likely to be on treatment with these drugs [20]. However, a study from the USA showed an almost 40% reduction in the risk of hospitalization among elderly patients with hypertension treated with ACEIs but not among those treated with ARBs [21]. The recommendation of both the American Heart Association and the European Society for Cardiology is to sustain treatment with these drugs in patients on them [22, 23]. This is also the recommendation of the French Society of Rheumatology [17].

### Statement 10

*Patients with stable RMDs should have their vaccination against influenza and pneumococci updated.*

The administration of influenza and pneumococcal vaccines to stable patients without COVID-19 was generally agreed upon. While it is not known if being vaccinated against influenza could reduce the severity of COVID-19, certainly, reducing this risk may cut down the potential confusion with COVID-19 among some patients [2, 24]. The task force recognized that national policies on vaccination differ among the member countries of AFLAR; members however agree that the ongoing pandemic should not reduce the use and administration of these vaccines for eligible patients.

### Statement 11

*Rheumatologists should reduce patients’ hospital attendances by considering less frequent blood monitoring for DMARDs among stable patients, longer prescription periods and virtual clinics.*

Wherever routine blood monitoring for DMARDs requires the attendance of patients to the hospital, reducing the frequency of laboratory investigations among stable patients will likely be of benefit [3]. This may imply re-organizing services and optimizing the line of communication between the healthcare service and the patient. Due to the increasing recognition of in-hospital transmission of SARS-CoV-2, limiting attendance will likely serve to reduce the anxiety of the healthcare workers and the patients [18]. As many patients with RMDs also have co-morbidities that put them at higher risk of severe COVID-19 such as metabolic syndrome, cardiovascular diseases and advanced age, it is important to explain this risk to patients and help them understand the purpose of reducing face-to-face interactions [25].

### Statement 12

*RMD patients on glucocorticoids who have been exposed to SARS-CoV-2 should continue their glucocorticoids at the lowest effective dose for maintaining disease control.*

Recognizing the risk of adrenal suppression and the uncertain benefit of aggressive steroid tapering or withdrawal led to the task force agreeing on sustaining glucocorticoid treatments albeit at doses not more than required to keep treated conditions controlled. This seems to align with the recommendations of other international associations of rheumatology [2–4, 17].

### Statement 13

*In patients who have been exposed to SARS-CoV-2 but are asymptomatic, continue anti-osteoporosis treatments including vitamin D and calcium supplementation.*

The task force noted that the widespread disruption of service may put osteoporosis service on a lower rung of priority than usual. In situations like this, efforts should be made, and patients are advised to sustain the ongoing use of osteoporosis prophylaxis or treatment in order to prevent the potentially devastating impacts of fragility fractures [26]. While COVID-19 poses a veritable threat to the African population, the management of other chronic health conditions should not suffer unintended consequences such as prolonged neglect resulting in mounting morbidity and mortality [27]. Hospital-administered treatments such as intravenous zolendronate and the training of self-injection of subcutaneous agents such as denosumab and teriparatide may face delays. It is not known for certain how long a delay of each agent is acceptable, but rheumatologists should assist the patients in preventing long treatment gaps wherever possible.

### Statement 14

*Following exposure to SARS-CoV-2 infection, continue Pneumocystis jirovecii pneumonia (PJP) prophylaxis in a patient who is already on it.*

The appropriate use of PJP prophylaxis before the advent of the novel SARS-CoV-2 should not stop in the current pandemic. It is agreed that the potential confusion that may result from the presentations of both diseases can lead to avoidable clinical dilemmas especially as the impact of superimposed PJP on COVID-19 has not been determined. Anecdotal reports have now shown PJP pneumonia mimicking COVID-19, and recent guidelines have advocated the sustenance of PJP prophylaxis [2, 28].

### Statement 15

*Regardless of recent exposure to SARS-CoV-2, ACEIs and ARBs may be initiated or increased in a patient with RMD if indicated.*

Despite the fear that has been associated with the use of ACEIs and ARBs regarding SARS-CoV-2 infection, there has not been any evidence that the use of these drugs makes proven infection worse. The task force agrees that it is logical to sustain treatment with these drugs among patients who have ongoing indications for their use except if there are other clinical reasons for discontinuation. This is similar to the position of other professional rheumatology bodies in Europe and America [3, 17].

### Statement 16

*Regardless of recent exposure to SARS-CoV-2, intra-articular steroid administration may be undertaken if indicated with the use of appropriate personal protective equipment (PPE).*

Where indicated, there is no evidence to avoid administering intra-articular steroids for patients where this is the best option of care after weighing the risk of hospital attendance. However, standard procedures regarding the use of personal protective equipment should be observed to limit the risk of transmission of SARS-CoV-2 infection between healthcare professionals and patients where such procedures are deemed justified [18].

### Statement 17

*In an RMD patient with confirmed or presumed COVID-19, ongoing glucocorticoid treatment should not be stopped but should be maintained at the lowest effective dose.*

It is agreed that the abrupt cessation of ongoing long-term glucocorticoid treatment in patients who have been on it in the long term before developing COVID-19 serves to cause more problems than prevent unproven worse outcome. This is similar to the recommendation of the ACR and EULAR, and it was agreed that advising the patient in advance regarding the use of glucocorticoids in the event of developing COVID-19 will help to prepare them properly [2–4]. In addition, a new trial has shown that dexamethasone reduces 28-day mortality among patients receiving oxygen or on mechanical ventilation [29].

### Statement 18

*In an RMD patient with confirmed or presumed COVID-19, unless specifically indicated, routine stoppage of NSAIDs, analgesics, ACEIs or ARB is not required.*

Strictly speaking, the new diagnosis of COVID-19 should not be taken as an indication for discontinuation of analgesics, NSAIDs, ACEIs or ARBs. This recommendation is based on the lack of evidence of a worse outcome among patients on these treatments, where they are sustained throughout the management of their COVID-19 symptoms.

### Statement 19

*Each rheumatology service should consider providing a helpline number to patients to use or for their doctors to use to seek rheumatological advice should they develop COVID-19.*

Various patterns of anxieties have been shown in the recent African survey of rheumatologists and their perception of their patients [1]. Some of these can be allayed in the patient by keeping an open line of communication and maintaining easy and rapid access to reaching the rheumatologist helpline for advice. In this period, it will not be unexpected for patients to be unusually anxious, and this state may be helped by having reliable access to guidance regarding the management of their RMD treatments and queries around their RMDs [18].

### Statement 20

*There is currently no proof that SARS-CoV-2 infection is transmitted through breastfeeding; thus, breastfeeding may be continued while observing personal hygiene precautions if a lactating woman has tested positive for SARS-CoV-2 infection.*

The nucleic acid of the SARS-CoV-2 virus has not been detected in breast milk, and it stands reasonable that the unlikely risk of transmission is much lower than the known benefits of continued breastfeeding. However, observation of standard infection-prevention practices should be advised [30]. Rooming-in may be feasible, and direct breastfeeding is advisable in a woman with suspected or confirmed COVID-19 [31]. The World Health Organization recommends continuing breastfeeding for as long as possible regardless of a mother’s COVID-19 status while practicing standard hygiene methods and taking respiratory precautions including wearing a mask [13].

### Statement 21

*In the event that a lactating mother with COVID-19 is too unwell to breastfeed the child, expressed breast milk or formula feeds should be considered, and these should be prepared using hygienic practices as recommended by government and regulatory bodies of the given region.*

Where the clinical status of the mother will make direct breastfeeding impractical, expressed breast milk is agreed on as an ideal feeding option, and this will not require pasteurization [31]. Formula feeding is an acceptable alternative if breastfeeding is not possible. It was recognized that preventing women in rural Africa from breastfeeding their children is likely to be associated with significant deleterious economic and health impacts and there is currently no evidence to advise exercising caution in this regard as long as hygienic practices are followed.

### Statement 22

*Among paediatric patients with suspected SARS-CoV-2 infection who have negative nasopharyngeal PCR test, PCR test on stool samples should be considered to help confirm the diagnosis.*

It may well be that relying only on the nasopharyngeal swab test for the diagnosis of COVID-19 in children will potentially lead to significant under-diagnosis, and, as such, it is agreed that faecal PCR test may be an additional screening method. Indeed, emerging data seem to suggest the possibility of faecal transmission as an additional route [32]. This may be from environmental contamination, a risk that is likely higher in both rural African communities and in urban slums. As children of all ages have been shown to develop COVID-19 without any gender disparity, healthcare professionals would do well to not exercise prolonged inertia in embarking upon appropriate testing of these patients, but it is advisable to limit the number of parents or family members attending with the patient to the barest minimum [18].

## Discussion

In the unprecedented experience of a world grappling with COVID-19, the task force was faced with a need to deliver a project backed by a limited amount of high-quality evidence. Certainly, this is a circumstance which rheumatology in Africa and beyond is not familiar with and which the rest of the world is currently struggling with as well. Preliminary preparation in the run up to setting out with this project included the consideration for commissioning a research and development company to conduct a systematic literature review of evidence in the shortest time possible. It soon became clear that the scanty amount and low quality of existing evidence on various relevant clinical questions as well as the lengthy duration needed for its execution made it an unworthy venture to proceed with. While a number of useful insights were drawn from the recommendations of national and international rheumatology societies outside Africa, aspects that are specific to paediatric rheumatology were excluded in most of these [2–4, 17]. However, the structure of AFLAR and the gradually growing membership of the PAFLAR usefully identified the need to work together to produce a common document in view of potential substantial overlaps in the relevant aspects of recommendation for adults and children in the era of COVID-19. The other peculiarity is the wide disparity in the structures of rheumatology and the entire healthcare services of the various countries covered by AFLAR.

A recognition of these variations meant that some flexibility is required with interpretation of some of these recommendations in the context of the prevailing local policies. Similarly, the relevance of some of the recommendations may succumb to the limit of available options of DMARDs and their formulations in some countries. Furthermore, the standard vaccination practices in some countries may dictate a contextual interpretation of the recommendation on influenza and pneumococcal vaccinations as provided in this document. It is recognized that with the emergence of more high-quality data over time, the need for current recommendations to evolve is acknowledged, and subsequent updated versions of these preliminary recommendations will be produced.

In its history, this is the first time that the AFLAR convened an emergency task force to deliver a formal draft of recommendations within a very short time in circumstances that have not been experienced by any member of the task force before. The virtual convening, execution and delivery of the project with seamless efficacy within the limit of resources and published evidence, however, signal a future of more possibilities in executing daunting tasks under difficult circumstances and tight schedules. Whereas there are still unanswered questions about the management of RMDs in the world of COVID-19, we identify that there are endless needs for research and service improvement to deliver the best care to the African patients. The task force agreed to target rheumatologists and not policy-makers, other physicians or patients directly. However, relevant aspects of advice suitable for patients are directed towards the rheumatologist for delivery to the African patients.

Certainly, patient education is crucial in the proper delivery of the safety practices and best approaches to dealing with RMDs in this precarious time of the pandemic. The coalition of national rheumatology societies within AFLAR will be a cornerstone in cascading the preliminary recommendations through their networks and working together to deliver subsequent updates with even more answers to clinical questions of wider pan-African spread. The immediate future calls for a continuation of seeking answers to various questions around the care for RMDs in the world of COVID-19, and it has become imperative that the lessons learnt so far be thoughtfully processed going forward.

## Data Availability

The data are available through the corresponding author at reasonable request
